# Boron microlocalization in oral mucosal tissue: implications for boron neutron capture therapy

**DOI:** 10.1054/bjoc.2000.1148

**Published:** 2000-06

**Authors:** G M Morris, D R Smith, H Patel, S Chandra, G H Morrison, J W Hopewell, M Rezvani, P L Micca, J A Coderre

**Affiliations:** Research Institute, University of Oxford, Churchill Hospital, Oxford, OX3 7LJ, UK; Department of Chemistry and Chemical Biology, Baker Laboratory, Cornell University, Ithaca, NY 14853–1301, USA; Medical Department, Brookhaven National Laboratory Upton, NY 11973, USA

**Keywords:** borocaptate sodium, p-boronophenylalanine, rat ventral tongue mucosa, compound biological effectiveness factor, ion microscopy imaging

## Abstract

Clinical studies of the treatment of glioma and cutaneous melanoma using boron neutron capture therapy (BNCT) are currently taking place in the USA, Europe and Japan. New BNCT clinical facilities are under construction in Finland, Sweden, England and California. The observation of transient acute effects in the oral mucosa of a number of glioma patients involved in the American clinical trials, suggests that radiation damage of the oral mucosa could be a potential complication in future BNCT clinical protocols, involving higher doses and larger irradiation field sizes. The present investigation is the first to use a high resolution surface analytical technique to relate the microdistribution of boron-10 (^10^B) in the oral mucosa to the biological effectiveness of the ^10^B(n,α)^7^Li neutron capture reaction in this tissue. The two boron delivery agents used clinically in Europe/Japan and the USA, borocaptate sodium (BSH) and p-boronophenylalanine (BPA), respectively, were evaluated using a rat ventral tongue model. ^10^B concentrations in various regions of the tongue mucosa were estimated using ion microscopy. In the epithelium, levels of ^10^B were appreciably lower after the administration of BSH than was the case after BPA. The epithelium:blood ^10^B partition ratios were 0.2:1 and 1:1 for BSH and BPA respectively. The ^10^B content of the lamina propria was higher than that measured in the epithelium for both BSH and BPA. The difference was most marked for BSH, where ^10^B levels were a factor of six higher in the lamina propria than in the epithelium. The concentration of ^10^B was also measured in blood vessel walls where relatively low levels of accumulation of BSH, as compared with BPA, was demonstrated in blood vessel endothelial cells and muscle. Vessel wall:blood ^10^B partition ratios were 0.3:1 and 0.9:1 for BSH and BPA respectively. Evaluation of tongue mucosal response (ulceration) to BNC irradiation indicated a considerably reduced radiation sensitivity using BSH as the boron delivery agent relative to BPA. The compound biological effectiveness (CBE) factor for BSH was estimated at 0.29 ± 0.02. This compares with a previously published CBE factor for BPA of 4.87 ± 0.16. It was concluded that variations in the microdistribution profile of ^10^B, using the two boron delivery agents, had a significant effect on the response of oral mucosa to BNC irradiation. From a clinical perspective, based on the findings of the present study, it is probable that potential radiation-induced oral mucositis will be restricted to BNCT protocols involving BPA. However, a thorough high resolution analysis of ^10^B microdistribution in human oral mucosal tissue, using a technique such as ion microscopy, is a prerequisite for the use of experimentally derived CBE factors in clinical BNCT. © 2000 Cancer Research Campaign


*British Journal of Cancer* (2000) **82**(11), 1764–1771
© 2000 Cancer Research Campaign
DOI: 10.1054/ bjoc.2000.1148, available online at http://www.idealibrary.com on IDEAL®

### Boron microlocalization in oral mucosal tissue: implications for boron neutron capture therapy

**GM Morris[1], DR Smith[2], H Patel[1], S Chandra[2], GH Morrison[2], JW Hopewell[1], M Rezvani[1], PL Micca[3] and JA Coderre[3]**

[1]Research Institute, University of Oxford, Churchill Hospital, Oxford, OX3 7LJ, UK; [2]Department of Chemistry and Chemical Biology, Baker Laboratory, Cornell University, Ithaca, NY 14853–1301, USA; [3]Medical Department, Brookhaven National Laboratory Upton, NY 11973, USA

**Summary** Clinical studies of the treatment of glioma and cutaneous melanoma using boron neutron capture therapy (BNCT) are currently taking place in the USA, Europe and Japan. New BNCT clinical facilities are under construction in Finland, Sweden, England and California. The observation of transient acute effects in the oral mucosa of a number of glioma patients involved in the American clinical trials, suggests that radiation damage of the oral mucosa could be a potential complication in future BNCT clinical protocols, involving higher doses and larger irradiation field sizes. The present investigation is the first to use a high resolution surface analytical technique to relate the microdistribution of boron-10 ($^{10}$B) in the oral mucosa to the biological effectiveness of the $^{10}$B(n,α)$^{7}$Li neutron capture reaction in this tissue. The two boron delivery agents used clinically in Europe/Japan and the USA, borocaptate sodium (BSH) and p-boronophenylalanine (BPA), respectively, were evaluated using a rat ventral tongue model. $^{10}$B concentrations in various regions of the tongue mucosa were estimated using ion microscopy. In the epithelium, levels of $^{10}$B were appreciably lower after the administration of BSH than was the case after BPA. The epithelium:blood $^{10}$B partition ratios were 0.2:1 and 1:1 for BSH and BPA respectively. The $^{10}$B content of the lamina propria was higher than that measured in the epithelium for both BSH and BPA. The difference was most marked for BSH, where $^{10}$B levels were a factor of six higher in the lamina propria than in the epithelium. The concentration of $^{10}$B was also measured in blood vessel walls where relatively low levels of accumulation of BSH, as compared with BPA, was demonstrated in blood vessel endothelial cells and muscle. Vessel wall:blood $^{10}$B partition ratios were 0.3:1 and 0.9:1 for BSH and BPA respectively. Evaluation of tongue mucosal response (ulceration) to BNC irradiation indicated a considerably reduced radiation sensitivity using BSH as the boron delivery agent relative to BPA. The compound biological effectiveness (CBE) factor for BSH was estimated at 0.29 ± 0.02. This compares with a previously published CBE factor for BPA of 4.87 ± 0.16. It was concluded that variations in the microdistribution profile of $^{10}$B, using the two boron delivery agents, had a significant effect on the response of oral mucosa to BNC irradiation. From a clinical perspective, based on the findings of the present study, it is probable that potential radiation-induced oral mucositis will be restricted to BNCT protocols involving BPA. However, a thorough high resolution analysis of $^{10}$B microdistribution in human oral mucosal tissue, using a technique such as ion microscopy, is a prerequisite for the use of experimentally derived CBE factors in clinical BNCT. © 2000 Cancer Research Campaign

**Keywords**: borocaptate sodium; p-boronophenylalanine; rat ventral tongue mucosa; compound biological effectiveness factor; ion microscopy imaging

Boron neutron capture therapy (BNCT) is a targeted radiotherapeutic modality used for the treatment of brain tumours and melanoma in the USA, Europe and Japan. BNCT was extensively reviewed in two recent articles (Barth et al, 1999; Coderre and Morris, 1999). The targeting effectiveness of BNCT is dependent upon the preferential delivery of boron-10 ($^{10}$B) to the primary tumour and its metastatic spread. A beam of thermalized neutrons is used to ‘activate’ the $^{10}$B, with the resultant production of highly localized high linear energy transfer (LET) radiation. This is due to the capture of thermal neutrons by $^{10}$B with the resultant fission reaction ($^{10}$B + $^{1}$n→ $^{11}$B→ $^{7}$Li + $^{4}$He (α) + 2.79 MeV) giving rise to high energy charged particles with short trajectories in tissue (5–9 μm). The microlocation and differential accumulation of $^{10}$B will therefore have a critical bearing on the therapeutic outcome of BNCT, and also on the response of normal tissue in the irradiated volume. In the clinical situation, relatively high therapeutic ratios have been obtained enabling BNCT to be given as a single dose or a small number (maximum 4) of dose fractions (Hatanaka et al, 1994; Coderre et al, 1997).

Mucositis is a commonly observed side-effect during conventional radiotherapy for cancer of the head and neck. While acute radiation damage of the oral mucosa is not generally associated with the current treatment of gliomas using BNCT, a number of patients involved in the American clinical studies are now presenting with transient acute effects. The problem relates to individuals with tumours at the front of the brain, in proximity to mucosal tissue (Coderre et al, 1997). This finding suggests that damage to the oral mucosa could be a potential complication in future BNCT clinical protocols involving larger irradiation field sizes than those in current use or multiple fields.

The purpose of the present investigation was to evaluate the microdistribution of $^{10}$B in the oral mucosa, and relate this to the biological effectiveness of the $^{10}$B(n,α)$^{7}$Li neutron capture reaction in this tissue. A CAMECA IMS-3f ion microscope, which is essentially a direct imaging secondary ion mass spectrometer (Benninghoven et al, 1987), was used to analyse cryopreserved

*Received 25 March 1999*
*Revised 28 January 2000*
*Accepted 17 February 2000*

*Correspondence to:* GM Morris tissue sections of the rat ventral tongue. This same instrument was employed in a previous study (Smith et al, 1996) to assess $^{10}B$ microlocalization in a rat brain tumour model for BNCT. Ion microscopy is a trace surface analytical technique with parts per million (ppm) detection limits for $^{10}B$. In principal the CAMCA IMS-3f can produce images of all isotopes from H to U with high resolution. The optics of the ion microscope maintain the spatial integrity of an analyte in a sample, producing images of isotopic distributions which can be related to tissue morphology with a resolution comparable to a conventional light microscope. A major advantage of using the CAMCA IMS-3f ion microscope for tissue microdistribution studies is the ability to observe and quantify, in a single image, the concentration gradient of $^{10}B$ in multiple zones of tissue. The $^{10}B$ distribution in the epithelium, lamina propria and muscle of the oral mucosa can be viewed simultaneously within a 150 µm diameter field of view.

The rat ventral tongue oral mucosa model, originally developed by Morris and Coderre (Coderre et al, 1999; Morris et al, 1999), was used in the present study. It is essentially a scaled up version of the standard murine ventral tongue model used in radiobiological studies (e.g. Moses and Kummermehr, 1986; Dorr and Kummermehr, 1990). The larger tongue area of the rat facilitates the jigging and shielding procedures required for local BNC irradiation, thereby reducing the radiation burden to the head. In the present study two boron delivery agents, borocaptate sodium (BSH) and boronophenylalanine (BPA), were used in the evaluation of $^{10}B$ microdistribution in the tongue mucosa. The radiobiological findings related to BSH-mediated BNC irradiation are compared and contrasted with a previous report (Coderre et al, 1999) detailing the effects of BPA-mediated BNC irradiation on the same model.

### MATERIALS AND METHODS

All experimental procedures involved the use of male Fischer 344 rats aged 12 weeks (260–290 g). The animal studies were reviewed and approved by the Brookhaven National Laboratory (BNL) Institutional Animal Care and Use Committee. Rats were accommodated two to a cage in temperature controlled rooms and had free access to food and water. They were maintained in a controlled light/dark cycle, with lights on between 07:00 and 18:00 h.

Disodium mercaptoundecahydro-close dodecaborate (borocaptate sodium) containing 55.1% (certified weight percentage) $^{10}B$ with 95.8% enrichment (Centronic Ltd., Croydon, UK) was used as the boron delivery agent. Vials of BSH powder, sealed under nitrogen gas, were opened immediately prior to use. Dilution was in sterile normal saline (10 mg BSH $ml^{-1}$). Administration of the BSH solution was by intraperitoneal (i.p.) injection in a volume of 1.5 ml (55 mg $kg^{-1}$; 30 mg $^{10}B$ $kg^{-1}$). BPA was given i.p. in a fructose solution at a dose of 700 mg $kg^{-1}$ (34 mg $^{10}B$ $kg^{-1}$) as described previously (Coderre et al, 1994). Concentrations of $^{10}B$ in solutions of BSH and BPA were determined using direct current plasma atomic emission spectroscopy (DCP-AES) and prompt gamma analysis (Fairchild et al, 1986; Barth et al, 1991).

### Boron biodistribution

Boron concentrations in blood and tongue were measured using (DCP-AES) and prompt gamma spectrometry.

The microdistribution of boron in the ventral tongue mucosa was evaluated using ion microscopy. The ion microscopy analyses were performed using a CAMECA IMS-3f ion microscope, a direct imaging secondary ion mass spectrometer. The use of this instrument for BNCT-related microlocalization studies has been described previously (Smith et al, 1996). In the present study the ion microscope was operated in the positive secondary ion imaging mode with the samples biased to +4500 V. A mass-filtered $O_2^+$ primary ion beam accelerated to 10 keV was focused and adjusted to a nominal beam current of 120 nA, producing a stationary beam spot with a diameter of approximately 50 µm at the surface of the sample. For all analyses, the primary ion beam was raster scanned over 250 × 250 µm square regions. The rate of erosion of the tissue specimens was approximately 20 Å per second. Singly charged positive secondary ions, originating from the surface of the sample, were collected as a global (non-mass-filtered) image by the instrument's immersion lens and focused using the 150 µm transfer optics in conjunction with a 60 µm contrast aperture. The energy window of the mass spectrometer was centred and set to a maximum value of 130 eV. Energy and mass filtered secondary ion images were magnified and projected on a single microchannel plate/phosphor screen image detection assembly. The gain of the microchannel plate was set to 73% of maximum. Images were recorded directly from the image detection assembly using a Photometrics Ltd. CH220 charged-coupled device (CCD) liquid-cooled camera head equipped with a Thompson-CSF TH7882 CDA CCD camera digitized to 14 bits/pixel by a photometric camera controller. For each region of analysis, sequential ion images from $^{39}K$, $^{23}Na$, $^{10}B$, $^{12}C$, $^{24}Mg$ and $^{40}Ca$ were acquired with integration times of 0.2 s, 0.2 s, 2 min, 2 min, 30 s and 30 s respectively. The digitized ion images were analysed using Alice™ 2.4.0 image processing software for the Macintosh (Hayden Image Processing Group). Quantification of the boron images was carried out using an ion microscopy calibration approach for biological specimens (Ausserer et al, 1989;Smith et al, 1996). Estimates of the $^{10}B$ concentration in the ventral tongue mucosa were determined from pixel to pixel registration and ratio of the $^{10}B$ and $^{12}C$ images, assuming an 85% tissue water content.

Samples for ion microscopy imaging were cryogenically prepared to preserve the in situ distribution of $^{10}B$ and minimize redistribution artefacts at the microscopic level. Two rats were administered BSH at 30 min prior to anaesthesia and euthanasia. Tissue specimens were frozen in liquid isopentane cooled to –150°C in liquid nitrogen. Frozen sections, 4-µm-thick, were cut perpendicular to the ventral surface of the tongue using a Reichert HistoSTAT cryostat microtome operated at –20°C. Sections were taken in series to correlate the tissue morphology (optical autofluorescence microscopy) with $^{10}B$ distribution (ion microscopy). Sections prepared for optical imaging were mounted on glass microscope slides and stained with haematoxylin and eosin. Sections designated for ion microscopy imaging were mounted on high purity indium/silicon substrates, freeze-dried for 24 h and coated with a 400 Å thick layer of Au/Pd alloy to minimize sample charging during analysis.

### Dosimetry

Boron neutron capture (BNC) irradiations were carried out on the thermal beam port of the Brookhaven Medical Research Reactor

© 2000 Cancer Research Campaign

**Table 1** Thermal beam exposure times, blood $^{10}B$ content at the time of irradiation and physical absorbed doses for the various treatment groups. Errors indicate ± s.e.m.

| Thermal beam exposure time (MW min) | Blood $^{10}B$ content ($\mu g\ g)^{-1}$ | Thermal beam dose component (Gy) | $^{10}B(n,_{\alpha})^7Li$ dose component (Gy) | Total dose (Gy) |
|---|---|---|---|---|
| 7.5 | 43.6 ± 0.9 | 1.6 | 9.3 | 10.9 |
| 8.5 | 46.6 ± 3.0 | 1.9 | 11.2 | 13.1 |
| 9.5 | 41.5 ± 2.0 | 2.1 | 11.2 | 13.3 |
| 12.5 | 44.7 ± 2.3 | 2.7 | 15.9 | 18.6 |
| 14.0 | 44.3 ± 2.5 | 3.0 | 17.7 | 20.7 |
| 15.0 | 46.6 ± 4.2 | 3.3 | 19.8 | 23.1 |
| 16.5 | 43.3 ± 1.4 | 3.6 | 20.3 | 23.9 |

(BMRR). The dosimetry of the mixed radiation field, which consisted of thermal neutrons, fast neutrons, gamma rays and charged particles (α, $^7Li$, $^1H$, $^{14}C$) from the $^{10}B(n,\alpha)^7Li$ and $^{14}N(n,p)^{14}C$ neutron capture reactions were calculated using thermal neutron fluence data from bare and cadmium-covered gold foils and wires (secured to and inserted in the tongues of dead rats). A uniform distribution of 2.7% w/w nitrogen in tissue was assumed. Thermoluminescent dosimeters (TLD-700: Harshaw Chemical, Solon, OH, USA) attached to the tongue, were used to measure the gamma dose. The combined fast (>10 keV) neutron and gamma doses at the beam port were measured using paired tissue-equivalent (TE) plastic ionization chambers containing methane-based tissue equivalent gas, and a graphite chamber filled with carbon dioxide gas. The fast neutron dose rate was calculated by subtraction of the gamma dose rate from the total dose rate. Monte Carlo computation, normalized to the in-air neutron dosimetry, was used to estimate the fast neutron dose rate at the level of the ventral tongue mucosa.

The thermal neutron fluence at the surface of the tongue was $5.46 \times 10^9$ $cm^{-2}$ $s^{-1}$ with the reactor running at 1 MW power, using a 10.2 cm thick collimator with a 20 mm diameter aperture. The collimator was constructed of lithium carbonate (7.5%) dispersed in polyethylene. The physical dose rates (cGy/MW min) were 2.84 (per $\mu g^{10}B\ g^{-1}$) for the $^{10}B(n,\alpha)^7Li$ reaction; 9.9 for the fast neutron interaction with hydrogen $^1H(n,n)p$; 7.0 for the nitrogen neutron capture reaction ($^{14}N(n,p)^{14}C$); and 4.8 for the total gamma-ray component (from the beam and induced in tissue by the $^1H(n,\gamma)^2H$ reaction).

### Irradiations

Anaesthesia during irradiation was maintained by the i.p. injection of ketamine (54 mg $kg^{-1}$) and xylazine (9 mg $kg^{-1}$). The anaesthetized rat was positioned in a body radiation shield (Joel et al, 1990; Morris et al, 1994*a*) that ensured that the rat was securely supported during irradiation. The procedure for thermal beam and BNC irradiation has been described in detail previously (Coderre et al, 1999). Briefly, the tongue was secured with tape to the 20-mm diameter collimator and positioned to ensure uniform irradiation of the ventral surface.

BSH was administered by i.p. injection 30 min prior to thermal neutron irradiation. A 0.5 ml blood sample was taken immediately prior to irradiation and the boron content determined using prompt gamma analysis. On average, eight rats were irradiated per dose group. The various dose groups are listed in Table 1. Radiation reactions in the tongue were scored daily, under light carbon dioxide/oxygen anaesthesia. Dose response was quantified using ulceration as the end point.

The dose–effect curve for the incidence of ulceration were fitted using probit analysis. The dose required to produce a 50% incidence of ulceration ($ED_{50}$) was calculated from this curve. Errors indicate the standard error of the mean (± s.e.m.).

### RESULTS

### Boron biodistribution and microlocalization

Blood $^{10}B$ concentrations from BSH for the various dose groups, obtained immediately prior to irradiation, are given in Table 1. In an additional evaluation of boron biodistribution (six rats) using DCP-AES, levels of $^{10}B$ in blood, ventral tongue and dorsal tongue were 38.9 ± 1.8, 21.1 ± 0.4 and 17.1 ± 0.6 μg $g^{-1}$ respectively.

The microdistribution of $^{10}B$ in the ventral tongue mucosa was evaluated using ion microscopy. The distributions of BSH and BPA were compared and $^{10}B$ concentrations were determined in the epithelium, lamina propria and contiguous ventral tongue muscle. Additionally, the boron accumulation characteristics of BSH and BPA were compared in the walls of blood vessels.

An optical (autofluorescence) image of cryosectioned ventral tongue mucosa of a rat administered BPA is shown in Figure 1A. White arrows point to the smooth surface of the stratified squamous epithelium which is generally not cornified on the lower surface of the tongue. The dotted lines in panel 1A were drawn to delineate the boundaries of the epithelium (Ep), the connective tissue of the lamina propria (LP) and cross-sections of striated muscle (M). The basal layer of the epithelium lies along the boundary of the epithelium and the lamina propria. Cells which are removed from the surface of the epithelium are replaced by cells which move up from the basal layer. The basal layer is vulnerable to radiation and damage manifests itself in this region. The circle superimposed in the optical image is scaled to 150 μm in diameter and correlates with the region on the adjacent cryosection analysed with the ion microscope (Figure 1 B–D). Dotted lines in the ion images of $^{10}B$, $^{24}Mg$ and $^{12}C$ (Figure 1 B–D) aid in visualizing the histology of the oral mucosa. The white arrows in the ion images point to the smooth surface of the epithelium. The ratio of the white pixel density in the $^{10}B$ and $^{24}Mg$ ion images (Figure 1 B,C) to the white pixel density of the $^{12}C$ image (Figure 1D), is directly proportional to the total concentration of boron and magnesium respectively. The image of the tissue matrix, $^{12}C$, is used in the

 *© 2000 Cancer Research Campaign*

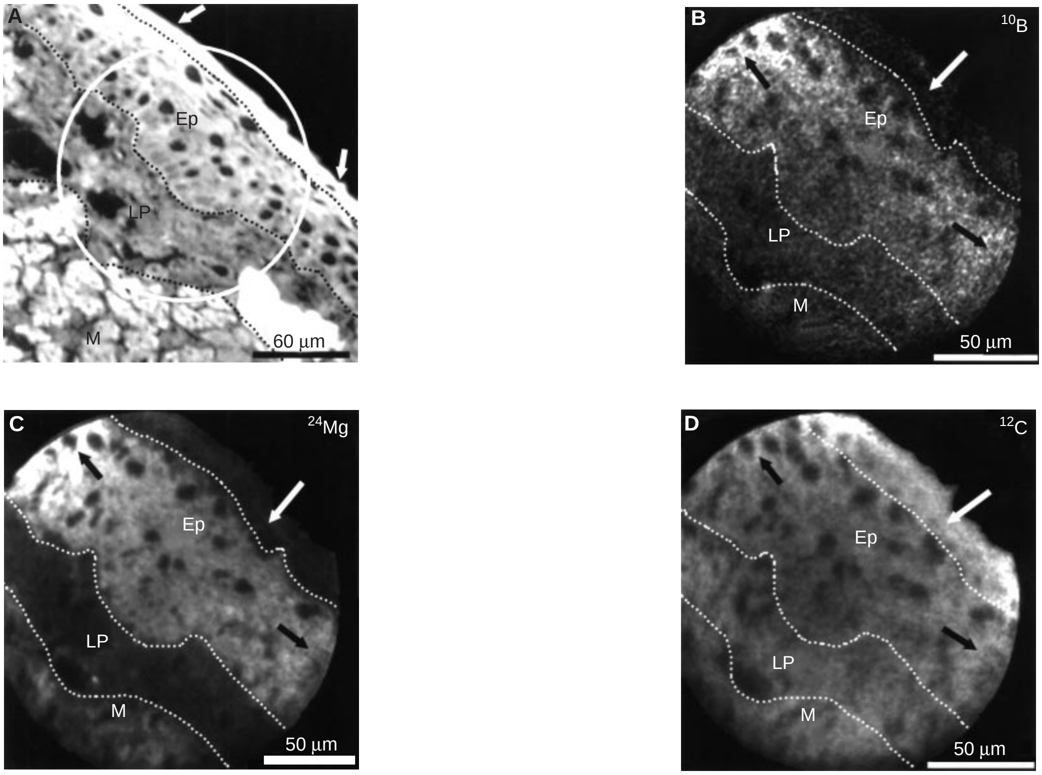

**Figure 1** Ventral tongue mucosa of a rat administered BPA. (**A**) Autofluorescence-fluorescein isothiocyanate (FITC) photomicrograph of haematoxylin and eosin stained cryosection of ventral tongue mucosa. The circle is scaled to 150 μm in diameter and correlates with the region of the adjacent cryosection of tissue analysed with the ion microscope (see panels **B–D**). (**B–D**) Sequential ion images from a freeze-dried cryosection of tissue mounted on indium showing the distribution of the isotopes $^{10}$B (**B**), $^{24}$Mg (**C**) and $^{12}$C (**D**). Images of $^{39}$K, $^{23}$Na and $^{40}$Ca are not shown. White arrows in (**A–D**) point to the outer surface of the epithelium. Black arrows in (**B–D**) point to regions of excessive brightness caused by the non-uniform response of the microchannel plate/phosphor screen detection assembly of the ion microscope. Dotted lines in (**A–D**) aid in visualization of the epithelium (Ep), lamina propria (LP) and muscle (M)

quantification scheme to reduce potential errors associated with the non-uniform response of the microchannel plate/phosphor screen detection assembly of the ion microscope (Ausserer et al, 1989). Temporal degradation of the detection assembly is responsible for the excessive brightness seen in the upper left and lower right quadrants of the $^{10}$B, $^{24}$Mg and $^{12}$C images (black arrows). In the absence of the $^{12}$C image, the white pixel intensity of the epithelium in Figure 1B,C would be incorrectly interpreted as a gradient with the highest concentrations of boron and magnesium in the upper left and lower right quadrants.

Not shown are images of total $^{39}$K, $^{23}$Na and $^{40}$Ca. These elements are imaged in sequence with $^{10}$B, $^{24}$Mg and $^{12}$C. Images of $^{39}$K, $^{23}$Na, $^{24}$Mg and $^{40}$Ca are relevant in assessing the physiological status of the cryopreserved tissue. The ratio of the image intensities of $^{39}$K to $^{23}$Na from regions within the epithelium and muscle were 3:1, indicating that the native distribution of these highly diffusible species was not significantly altered. The ratio of the image intensities of $^{39}$K to $^{23}$Na in the lamina propria falls off to nearly 1:1 which is consistent with an area rich in blood vessels. Blood has a relatively high sodium and low potassium content. The $^{24}$Mg ion image (Figure 1C) shows a gradient of distribution consistent with that observed for $^{39}$K and $^{23}$Na. The epithelium and muscle had a high Mg content while the lamina propria had low levels of Mg. As with K, the Mg content of blood is low relative to the vessels and surrounding tissue.

Calcification (not shown) was limited to a thin layer at the ventral surface of the tongue (white arrows in Figure 1 B,C) and some extracellular spaces, the latter possibly due to some form of calcium precipitate. Viable tissues were not excessively calcified, indicating minimal damage due to sample preparation.

Sequential ion images of $^{24}$Mg and $^{10}$B from two regions of the ventral tongue mucosa of a rat given BSH are shown in Figure 2. The distribution of $^{24}$Mg in the epithelium, lamina propria and muscle was similar to that observed after BPA administration. Mg was high in the epithelium and muscle and low in the lamina propria. Assessment of the physiological status of the cryopreserved tissue based on the estimated concentrations of K, Na, and Ca (images not shown) was also consistent with the pattern of distribution observed in the BPA-treated rats. However, the distribution pattern of $^{10}$B from BSH contrasts with that observed in the tongues of rats treated with BPA. There were significantly reduced concentrations of $^{10}$B in the epithelium and muscle relative to the lamina propria and blood. The estimated $^{10}$B concentrations in the various regions of the ventral tongue mucosa, after the administration of BSH and BPA are given in Table 2.

The values in Table 2 are reported as average $^{10}$B concentrations for the individual histological features and are not subcellular assignments. Although the CAMECA IMS-3f ion microscope is operated to produce ion images with a nominal resolution of ~0.5 μm, the concentration gradient of $^{10}$B within the histological features analysed was insufficient to resolve cellular boundaries.

Blood $^{10}$B levels were measured using DCP-AES and not ion microscopy. This is due to the fact that the blood components of arteries and vessels are often lost during cryogenic sample

© 2000 Cancer Research Campaign

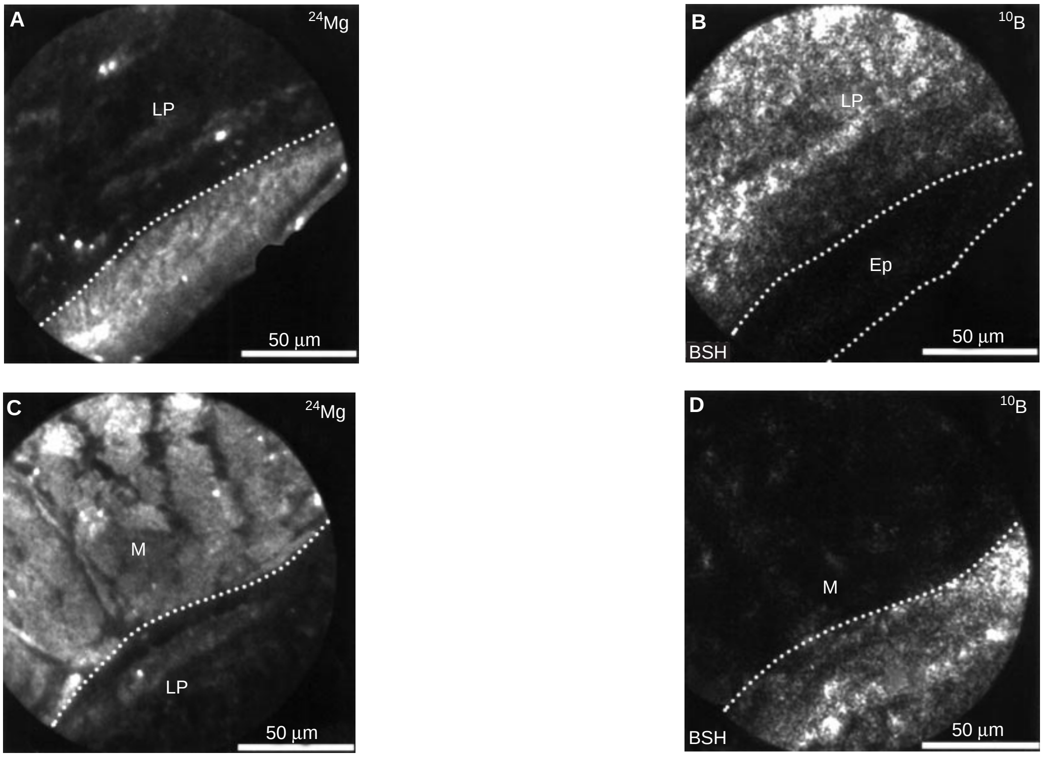

**Figure 2** Ventral tongue mucosa of a rat administered BSH. Panels **A, B** and panels **C, D** are sequential ion images from different regions of analysis of a freeze-dried cryosection of tissue on indium. (**A, C**) Ion images showing the distribution of the isotope $^{24}$Mg. (**B, D**) Ion images showing the distribution of the isotope $^{10}$B. Images of $^{12}$C, $^{39}$K, $^{23}$Na and $^{40}$Ca are not shown. Dotted lines in panels **A–D** aid in visualization of the Ep, LP and M. For key to symbols refer to Figure 1

**Table 2** Measurement of $^{10}$B concentrations in ventral tongue mucosa using ion microscopy based direct imaging secondary ion mass spectrometry (SIMS). Errors indicate ± s.e.m.

| Boron delivery agent | Epithelium µg g$^{-1}$ | Lamina propria µg g$^{-1}$ | Muscle µg g$^{-1}$ | Blood vessel wall µg g$^{-1}$ | Blood[a] µg g$^{-1}$ |
|---|---|---|---|---|---|
| BSH | 6.1 ± 1.0<br>[b] 5.8 ± 0.4 | 36.2 ± 4.2 | 6.6 ± 0.6 | 9.7 ± 1.3 | 36.6 ± 6.4 |
| BPA | 21.5 ± 0.5<br>[b]21.6 ± 0.4 | 31.1 ± 1.2 | 15.6 ± 1.7 | 19.2 ± 1.6 | 20.7 ± 0.7 |

[a] Blood $^{10}$B concentration was measured using DCP-AES. [b]Basal cell layer $^{10}$B concentration.

preparation for ion microscopy. The $^{10}$B concentration in the epithelium of BSH-treated rats was a factor of ~3.5 lower than that found in the epithelium of BPA-treated rats. $^{10}$B was distributed uniformly throughout the viable epithelium. Analysis of the basal layer, which is the location of the epithelial stem cells, indicated similar levels of $^{10}$B ($P > 0.1$, Student's *t*-test) to that measured in the entire viable epithelium (Table 2). The epithelium:blood $^{10}$B partition ratios were 0.2:1 and 1:1 for BSH and BPA respectively. In other words, levels of $^{10}$B in the epithelium were a factor of 5 lower than in the blood after BSH administration, but similar to those in the blood after BPA administration. The lamina propria:blood $^{10}$B partition ratio was 1:1 after the administration of BSH and is consistent with a relatively high density of blood vessels in this region. For BPA treated rats, the lamina propria:blood $^{10}$B partition ratio was 1.5:1. The reason for the higher levels of $^{10}$B in the lamina propria, as compared with the blood, is not entirely clear. It may be related to the much broader range of $^{10}$B concentrations obtained in the lamina propria after BPA (8–75 µg g$^{-1}$, 168 samples) as compared with BSH administration. The concentration of $^{10}$B in muscle was a factor of ~1.3 lower than that in the blood of rats given BPA, but were a factor of ~5 lower than the blood in rats given BSH. The quantity of $^{10}$B in blood vessel walls was relatively low (9.7 ± 1.3 µg g$^{-1}$) after BSH administration but was considerably higher (19.2 ± 1.6 µg g$^{-1}$) after BPA administration (Table 2).

### Radiation response

The mean $^{10}$B concentrations in the blood, measured at the time of irradiation, were used in the calculation of the physical radiation dose from the $^{10}B(n,\alpha)^{7}Li$ reaction. This resulted in a potential variation in the total physical dose (which included the thermal

 *© 2000 Cancer Research Campaign*

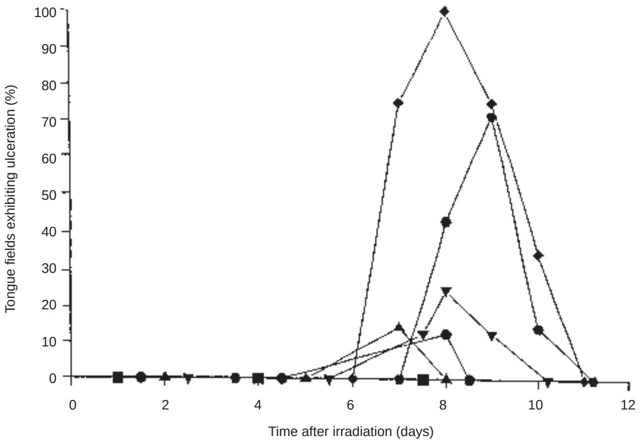

**Figure 3** Time-related changes in the incidence of tongue ulceration after thermal neutron irradiation in the presence of BSH. The doses shown are physical radiation doses. ■ = 10.9 Gy; ● = 13.1 Gy; ▲ = 13.3 Gy; ▼ = 18.6 Gy; ⬢ = 23.1 Gy; ◆ = 23.9 Gy

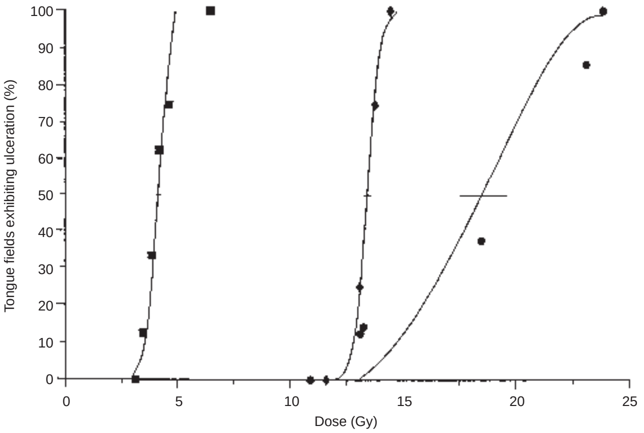

**Figure 4** Dose-related change in the incidence of tongue ulceration after irradiation with thermal neutrons in the presence of BSH. Dose–effect curves for the thermal beam-alone and X-rays are shown by way of comparison (Coderre et al, 1999). The doses shown are physical radiation doses ■ = thermal beam-alone; ◆ = X-rays; ● = thermal beam plus BSH

beam dose) of ≤ 7.6%. Irradiation times were short (3–5 min) because the BMR reactor was operated at full power (3 MW). As a result, fluctuation in the $^{10}B$ content of the blood was minimal over the period of irradiation.

Ulceration of the ventral surface of the tongue was apparent by 7–8 days after irradiation with thermal neutrons in the presence of BSH. The proportion of animals in which the tongue field exhibited ulceration was dose dependent (Figure 3). Peak incidence of ulceration was at 8–9 days after irradiation. Healing was rapid and was completed by 8–11 days after irradiation. The dose-related incidence of ulceration is shown in Figure 4. Previously published data (Coderre et al, 1999) using the identical end point and strain of rat are shown by way of comparison. $ED_{50}$ values for the ulceration end point were calculated from these curves. In defining the biological effects of the $^{10}B(n,\alpha)^7Li$ neutron capture reaction relative to photons, the term compound biological effectiveness (CBE) factor was used as an alternative to RBE. The rational behind this has been discussed in detail previously (Morris et al, 1994). Calculation of the CBE is similar to that of the RBE factor. Equating the X-ray $ED_{50}$ dose with a BNC dose (beam + BSH) that gives the same end point of a 50% incidenc of ulceration produces the following equation:

CBE factor = [(X-ray $ED_{50}$) – (thermal beam component of $ED_{50} \times$ RBE)]/$^{10}B(n,\alpha)^7Li$ component of $ED_{50}$.

In Figure 4 it is evident that the dose–effect curve for ulceration, after BSH-mediated BNC irradiation, is shifted to the far right indicating that this modality has a considerably reduced biological effectiveness as compared with the thermal beam-only irradiation modality or X-rays. Previously published $ED_{50}$ values (Coderre et al, 1999) for X-ray (13.4 ± 0.2 Gy) and thermal neutron beam (BMRR) irradiation (4.2 ± 0.1 Gy), together with the thermal beam RBE (3.2 ± 0.1), were used in the calculation of the CBE factor for BSH. These were obtained using the same biological end point in the identical strain, age and sex of rat to that used in the present study. The $ED_{50}$ for BSH mediated BNC irradiation was calculated at 18.6 ± 1.0 Gy. Of this value, 2.8 Gy was from the thermal beam and 15.8 Gy was from the $^{10}B(n,\alpha)^7Li$ reaction. Solving the above equation indicates a CBE factor for BSH of 0.29 ± 0.02.

### DISCUSSION

In the present study, the end point used to evaluate the effects of BNC irradiation on oral mucosa, was tongue ulceration. This is an acute effect caused by radiation induced sterilization and death of stem cells in the mucosal epithelium. Eventual denudation of the epithelium leads to ulceration. The epithelium is therefore the critical area for $^{10}B$ analysis in the tongue mucosa. The initial occurrence of tongue ulceration was at 7–8 days after irradiation with thermal neutrons in the presence of BSH. This compares with a latency to tongue ulceration of 6–8 days after irradiation with X-rays, thermal beam – alone or in combination with BPA – using the identical strain of rat (Coderre et al, 1999). For all irradiation modalities, the time period over which tongue ulceration was evident increased with increasing radiation dose. Comparable findings have been documented by others using murine models. In the case of ventral tongue, single exposures of 29 kV X-rays, at doses in excess of 15 Gy, resulted in the development of ulcers by days 9 and 10 (Moses and Kummermehr, 1986; Dorr and Kummermehr, 1990). Mucositis was evident in mouse lips at 9 to 10 days after single dose irradiation with 250 kV X-rays or $^{60}Co$ (Parkins et al, 1983; Ang et al, 1984). In the clinic, fractionation protocols involving irradiation over periods of 2 weeks or less have resulted in the appearance of mucositis at 9–10 days after the start of irradiation (Dutreix et al, 1971; Parkins et al, 1983; van der Schueren et al, 1983). This suggests that the turnover time of mucosal epithelium is comparable in the various rodent models and man.

The dose contribution from the $^{10}B(n,\alpha)^7Li$ reaction is routinely calculated on the basis of the blood $^{10}B$ concentration during the course of thermal/epithermal neutron irradiation. No account is taken of the $^{10}B$ content of critical normal tissues in the irradiation field in the dose calculations. This is because, at present, it is not possible to estimate normal tissue concentrations of $^{10}B$ during the course of BNC irradiation. Non-invasive imaging techniques are currently under development in an attempt to achieve this objective (e.g. Kabalka et al, 1997; Imahori et al, 1998). The physical dose delivered to a specific normal tissue is therefore described in terms of the physical dose delivered to the blood. As a consequence, the calculated CBE factor will be strongly

© 2000 Cancer Research Campaign influenced by variations in the amount of $^{10}B$ present in the target normal tissue relative to the blood (i.e. the $^{10}B$ partition ratio). In the present study, based solely on the $^{10}B$ concentration of the blood, the CBE factor for BSH was estimated at ~0.3. This compares with a CBE estimate for BPA of ~4.9, using the identical strain, sex and age of rat (Coderre et al, 1999).

RBE and CBE factors have been reported for skin, using moist desquamation (epithelial denudation) as the end point. For thermal beams, RBE values in the range 2.7–3.9 have been documented (Yamamoto, 1961; Archambeau, 1989; Morris et al, 1994). A CBE factor of ~2.5 has been estimated for both hamster and human skin using BPA as the boron delivery agent (Hiratsuka et al, 1991; Fukuda et al, 1994). A higher CBE estimate for BPA of ~3.7 was reported using rat skin (Morris et al, 1994). The strain, age and sex were identical to that employed in the present study. This suggests that the oral mucosa (CBE ~4.9) is more sensitive to BPA-mediated BNC irradiation than the skin. With BSH the CBE factor for tongue mucosa was much lower at ~0.3. This value is comparable with analogous CBE estimates (~0.4–0.5) for rat and dog skin (Morris et al, 1994; Gavin et al, 1997).

Ion microscopy imaging studies of $^{10}B$ in body tissues (Smith et al, 1996, 1997), has enabled the biodistribution of this element to be analysed with considerably greater precision than was previously possible. Although other analytical techniques have been utilized for boron biodistribution studies, such as quantitative neutron capture autoradiography (Gabel et al, 1987*a*) or the electron microprobe (Feldman and Mayer, 1986), they lack either the spatial resolution or the sensitivity necessary to determine the microdistribution of this trace analyte. Ion microscopy is providing new insights into the relationship between $^{10}B$ biodistribution and the biological effectiveness of the high LET particles produced by the $^{10}B(n,_{\alpha})^{7}Li$ neutron capture reaction in vivo. Bulk analysis (DCP-AES) of whole tissue samples from the ventral tongue mucosa indicated appreciable uptake of $^{10}B$ (~21 μg $g^{-1}$) after the administration of BSH. A similar finding was reported for BPA administered at a comparable $^{10}B$ concentration to BSH (Coderre et al, 1999), where the level of $^{10}B$ in the ventral tongue was estimated at ~23 μg $g^{-1}$. While this data indicated that the overall concentrations of $^{10}B$ in the tongue were similar for both delivery agents, it provided no information with regard to the biodistribution profile of the $^{10}B$ in different regions of the tissue. Ion microscopy analysis revealed that the level of $^{10}B$ in the mucosal epithelium was very low after BSH administration. Indeed, the $^{10}B$ content of the mucosal epithelium was ~3.5 times higher in rats treated with BPA. In the case of BSH, the majority of the $^{10}B$ was located in the lamina propria. This differs from the findings for BPA where the $^{10}B$ content in the mucosal epithelium was closer to that in the lamina propria, the differentials being 1:6 for BSH and 1:1.5 for BPA. Mucosal epithelium:blood $^{10}B$ partition ratios were ~0.2 (6.2:36.6) and ~1 (21.5:20.7) for BSH and BPA respectively. In other words, $^{10}B$ accumulation in the epithelium, relative to the blood, was 5 times higher with BPA than with BSH. The physical dose (the blood dose) that resulted in a 50% incidence ($ED_{50}$) of ventral tongue ulceration was ~3.0 Gy using BPA (Coderre et al, 1999) and ~18.6 Gy using BSH. When the total radiation dose that was delivered directly to the mucosal epithelium was estimated from the ion microscopy-derived $^{10}B$ concentrations, it was ~3.2 Gy and ~4.9 Gy for BPA and BSH respectively. The CBE factor values calculated from these $ED_{50}$s were ~4.6 for BPA and ~2.2 for BSH. This indicates that a major factor contributing to the difference in the CBE values (0.3 versus 4.9), estimated using blood $^{10}B$ concentrations, was low uptake of BSH by the mucosal epithelium. However, the fact that even taking into account the $^{10}B$ levels in the epithelium in radiation dose calculations, the CBE factor for BPA was a factor of ~2 higher than for BSH suggests that the microdistribution of $^{10}B$ from these two compounds differs at the intracellular level.

The majority of the energy released after the capture of a thermal neutron by a $^{10}B$ atom ($^{10}B + n \rightarrow {}^{11}B \rightarrow {}^{7}Li + \alpha + 2.79$ MeV) is carried by the α and $^{7}Li$ charged particles. Thus, as a consequence, the pattern of energy deposition in critical cell organelles, such as the nucleus is strongly influenced by the microdistribution of the $^{10}B$ atoms. Monte Carlo simulations have demonstrated that $^{10}B$ located in the cell nucleus is more effective than $^{10}B$ distributed in the cytoplasm, which is in turn more effective than boron attached to the cell membrane (Kobayashi and Kanda, 1982; Gabel et al, 1987*b*; Verrijik et al, 1994). Thus, the microlocalization of $^{10}B$ has a critical effect on the biological effectiveness of the $^{10}B(n,\alpha)^{7}Li$ reaction. Monte Carlo simulations for a cubic cell with a volume of 1150 $\mu m^3$ and a spherical nucleus of volume 230 $\mu m^3$ (representative of a basal epithelial cell) indicate a sevenfold reduction in efficacy when the $^{10}B$ is located exclusively extracellularly, and a fivefold increase in efficacy for $^{10}B$ is located within the nucleus, as compared with the same amount of $^{10}B$ distributed homogeneously (Gabel et al, 1987*b*). At the present time, data is not available on the precise microlocation of $^{10}B$ at the intracellular level for the mucosal epithelium. While the resolution (~0.5 μm) of the ion microscope is sufficiently high to evaluate the subcellular localization of $^{10}B$, it is not possible at present to delineate the boundaries of individual epithelial cells in tissue sections. For this reason, the subcellular microdistribution of $^{10}B$ was not evaluated in the current study. The data presented details the average concentration of $^{10}B$ in the various histological zones of the tongue mucosa, such as the mucosal epithelium. More specific evaluation of the basal layer (the location of the stem cells) revealed no appreciable difference in $^{10}B$ content relative to the higher suprabasal layers in the epithelium. This was true for both BSH and BPA.

### CONCLUSIONS

The ventral tongue mucosa of the rat was considerably less sensitive to BSH as compared with BPA-mediated BNCT. This reduced sensitivity was directly related to the microdistribution profile of $^{10}B$. At similar doses of $^{10}B$ from BSH or BPA, the concentration of this element was a factor of ~3.5 lower in the mucosal epithelium after BSH as compared with BPA administration. At the present time, due to practical considerations, CBE factors are estimated on the basis of the radiation dose from the $^{10}B(n,\alpha)^{7}Li$ neutron capture reaction delivered to the blood. While this is a valid approach, experimentally derived CBE factors from animal models must be used in BNCT clinical dosimetry protocols with extreme caution. The microdistribution profiles of $^{10}B$, for a given boron delivery agent in a given tissue, must be comparable in the relevant animal model and man for reliable extrapolation of the CBE factor.

Bulk analytical techniques, such as prompt gamma and DCP-AES are routinely used for the intercomparison of $^{10}B$ biodistribution in human and animal tissues. The findings of the present study indicate that such an approach is inadequate, because no information is provided on $^{10}B$ accumulation in critical target cell compartments. A major advance is offered by ion microscopy, which facilitates the simultaneous analysis of $^{10}B$ microdistribution and

*© 2000 Cancer Research Campaign*

concentration at high resolution in individual tissue compartments, such as the mucosal epithelium.

### ACKNOWLEDGEMENTS

This work was supported by the Cancer Research Campaign. Additional support was provided by the Office of Biological and Environmental Research, US Department of Energy, under contract number DE-AC02-98CH10886 and DE-FG02-ER61138.

### REFERENCES

Ang KK, Landuyt W, Rijners A and Schueren and van der E (1984) Differences in repopulation kinetics in mouse skin during split course multiple fractions per day (MDF) or daily fractionated irradiations. *Int J Radiat Oncol Biol Phys* **10**: 95–99

Archambeau JO (1989) A model to evaluate dose recovery from different radiation. In: *Clinical Aspects of Neutron Capture Therapy*, pp. 9–20. Plenum Press: New York

Ausserer WA, Ling YC, Chandra S and Morrison GH (1989) Quantitative imaging of boron, calcium, magnesium, potassium and sodium distributions in cultured cells with ion microscopy. *Anal Chem* **61**: 2690–2695

Barth RF, Adams DM, Soloway AH, Mechetner EB, Alam F and Anisuzzaman AKM (1991) Determination of boron in tissues and cells using direct current plasma atomic emission spectroscopy. *Anal Chem* **63**: 890–893

Barth RF, Soloway AH, Goodman JH, Gahbauer RA, Gupta N, Blue TE, Yang W and Tjarks W (1999) Boron neutron capture therapy of brain tumours: an emerging therapeutic modality. *Neurosurgery* **44**: 433–451

Benninghoven A, Rudenauer FG and Werner HW (1987) Instrumentation In: Elving PJ, Eincfordner JD and Kolthoff IM (Eds) Secondary ion mass spectrometry, basic concepts, instrumental aspects, applications and trends, pp. 594–606. John Wiley & Sons: New York

Coderre JA and Morris GM (1999) The radiation biology of boron neutron capture therapy. *Radiat Res* **151**: 1–18

Coderre JA, Button TM, Micca PL, Fisher CD, Nawrocky MM and Liu HB (1994) Neutron capture therapy of the 9L rat gliosarcoma using the p-boronophenylalanine–fructose complex. *Int J Radiat Oncol Biol Phys* **30**: 643–652

Coderre JA, Bergland R, Capala J, Chadha M, Chanana AD, Elowitz E, Joel DD, Liu HB and Slatkin DN (1997) Boron neutron capture therapy for glioblastoma multiform using p-boronophenylalanine and epithermal neutrons: trial design and early clinical results. *J Neuro-oncol* **33**: 141–152

Coderre JA, Morris GM, Micca PL, Ma RM and Gordon CR (1999) The effects of boron neutron capture irradiation on oral mucosa: evaluation using a rat tongue model. *Radiat Res* **152**: 113–118

Dorr W and Kummermehr J (1990) Accelerated repopulation of mouse tongue epithelium during fractionated irradiations or following single doses. *Radiother Oncol* **17**: 249–259

Dutreix J, Tubiana M, Wambersie A and Malaise E (1971) The influence of cell proliferation in tumours and normal tissues during fractionated radiotherapy. *Eur J Cancer* **7**: 205–213

Fairchild RG, Gabel D, Laster BH, Greenberg D, Kiszenick W and Micca PL (1986) Microanalytical techniques for boron analysis using the $^{10}B(n,\alpha)^7Li$ reaction. *Med Phys* **13**: 50–56

Feldman LC and Mayer JW (1986) Radiative transitions and the electron microprobe. In: *Fundamentals of Surface and Thin Film Analysis*, pp. 243–247. North Holland: New York

Fukuda H, Hiratsuka J, Honda C, Kobayashi T, Yoshino K, Karashima H, Takahashi J, Abe Y, Kanda K, Ichihashi M and Mishima Y (1994) Boron neutron capture therapy of malignant melanoma using $^{10}B$-paraboronophenylalanine with special reference to evaluation of radiation dose and damage to the skin. *Radiat Res* **138**: 435–442

Gabel D, Holstein H, Laarsson B, Gille L, Ericson G, Sacker D, Som P and Fairchild RG (1987*a*) Quantitative neutron capture radiography for studying the biodistribution of tumour-seeking boron-containing compounds. *Cancer Res* **47**: 5451–5454

Gabel D, Foster S and Fairchild RG (1987*b*) The Monte Carlo simulation of the biological effect of the $^{10}B(n,_{\alpha})^7Li$ reaction in cells and tissue and its implications for boron neutron capture therapy. *Radiat Res* **111**: 14–25

Gavin PR, Kraft SL, Huiskamp R and Coderre JA (1997) A review: CNS effects and normal tissue tolerance in dogs. *J Neuro-Oncol* **33**: 71–80

Hatanaka H and Nakagawa Y (1994) Clinical results of long-surviving brain tumour patients who underwent boron neutron capture therapy. *Int J Radiat Oncol Biol Phys* **28**: 61–66

Hiratsuka J, Fukuda H, Kobayashi T, Karashima H, Yoshino K, Imajo Y and Mishima Y (1991) The relative biological effectiveness of $^{10}B$ neutron capture therapy for early skin reactions in the hamster. *Radiat Res* **128**: 186–191

Imahori Y, Ucda Y, Ohmori T, Kusuki T, Ono K, Fujii R and Ido T (1998) Fluorine-18 labelled fluoroboronophenylalanine PET in patients with glioma. *J Nucl Med* **39**: 325–333

Joel DD, Fairchild RG, Laissue JA, Saraf SK, Kalef-Ezra JA and Slatkin DN (1990) Boron neutron capture therapy for intracerebral rat gliosarcomas. *Proc Natl Acad Sci USA* **87**: 9808–9812

Kabalka GW, Smith GT, Dyke JP, Reid WS, Longford CPD, Roberts TG, Reddy NK, Buonocore E and Hubner KFC (1997) Evaluation of fluorine-18 BPA fructose for boron neutron capture treatment planning. *J Nucl Med* **38**: 1762–1767

Kobayashi T and Kanda K (1982) Analytical calculation of boron-10 dosage in cell nucleus for neutron capture therapy. *Radiat Res* **91**: 77–94

Morris GM, Coderre JA, Hopewell JW, Micca PL and Rezvani M (1994) Response of rat skin to boron neutron capture therapy with p-boronophenylalanine or borocaptate sodium. *Radiother Oncol* **39**: 253–259

Morris GM, Coderre JA, Smith DR and Hopewell JW (1999) A rat model of oral mucosal response to boron neutron capture irradiation. In: *Proceedings of the Eighth International Symposium on Neutron Capture therapy for Cancer*. Plenum Press: New York (in press).

Moses R and Kummermehr J (1986) Radiation response of the mouse tongue epithelium. *Brit J Cancer* **53**: Suppl VII, 12–15

Parkins C, Fowler JF and Shen Y (1983) A murine model of lip epidermal/mucosal reactions to x-irradiation. *Radiother Oncol* **1**: 159–165

Smith DR, Chandra S, Coderre JA and Morrison GH (1996) Ion microscopy imaging of $^{10}B$ from p-boronophenylalanine in a brain tumour model for boron neutron capture therapy. *Cancer Res* **56**: 4302–4306

Smith DR, Chandra S, Coderre JA, Barth RF, Yang W, Liu L, Wilson JL, Micca PL, Nawrocky MM, Rotaru JH and Morrison GH (1997) Quantitative ion microscopy imaging of boron-10 in rat brain tumour models for BNCT. In: *Advances in Neutron Capture Therapy. Vol. II, Chemistry and Biology*, Larson B, Crawford J and Weinreich R pp. (eds), 308–314. Elsevier Science: Amsterdam.

van der Schueren E, van der Bogaert W and Ang KK (1983) Radiotherapy with multiple fractions per day. In: *The Biological Basis of Radiotherapy*, Steel GG, Adams G and Peckam M (eds), pp. 195–210. Elsevier Science: Amsterdam

Verrijk R, Huiskamp R, Begg AC, Wheeler FJ and Watkins PRD (1994) A comprehensive PC based computer model for microdosimetry of BNCT. *Int J Radiat Biol* **65**: 241–253

Yamamoto YL (1961) The biological effectiveness of thermal neutrons and of the heavy particles from the $^{10}B(n,\alpha)^7Li$ reaction for the rabbits ear and its utilisation for neutron capture therapy. *Yokohoma Med Bull* **12**: 4–22

*© 2000 Cancer Research Campaign* 
